# Reduced Number of Pediatric Orthopedic Trauma Requiring Operative Treatment during COVID-19 Restrictions: A Nationwide Cohort Study

**DOI:** 10.1177/1457496920968014

**Published:** 2020-10-26

**Authors:** A. Raitio, M. Ahonen, M. Jääskelä, J. Jalkanen, T. T. Luoto, M. Haara, Y. Nietosvaara, A. Salonen, N. Pakkasjärvi, T. Laaksonen, J. J. Sinikumpu, J. Syvänen

**Affiliations:** 1 Department of Pediatric Surgery and Orthopedics, University of Turku, Turku University Hospital, Turku, Finland; 2 Department of Pediatric Surgery and Orthopedics, Helsinki Children’s Hospital, Helsinki, Finland; 3 Department of Pediatric Surgery and Orthopedics, Oulu University Hospital and PEDEGO Research Unit, University of Oulu, Oulu, Finland; 4 Department of Pediatric Surgery, Kuopio University Hospital, Kuopio, Finland; 5 Department of Pediatric Surgery, Tampere University Hospital, Tampere, Finland

**Keywords:** COVID-19, coronavirus, fractures, pandemic, pediatric orthopedics, pediatric surgery

## Abstract

**Background and Aims:**

The coronavirus outbreak significantly changed the need of healthcare services. We hypothesized that the COVID-19 pandemic decreased the frequency of pediatric fracture operations. We also hypothesized that the frequency of emergency pediatric surgical operations decreased as well, as a result of patient-related reasons, such as neglecting or underestimating the symptoms, to avoid hospital admission.

**Materials and Methods:**

Nationwide data were individually collected and analyzed in all five tertiary pediatric surgical/trauma centers in Finland. Operations related to fractures, appendicitis, and acute scrotum in children aged above 16 years between March 1 and May 31 from 2017 to 2020 were identified. The monthly frequencies of operations and type of traumas were compared between prepandemic 3 years and 2020.

**Results:**

Altogether, 1755 patients were identified in five tertiary hospitals who had an emergency operation during the investigation period. There was a significant decrease (31%, *p* = 0.03) in trauma operations. It was mostly due to reduction in lower limb trauma operations (32%, *p* = 0.006). Daycare, school, and organized sports–related injuries decreased significantly during the pandemic. These reductions were observed in March and in April. The frequencies of appendectomies and scrotal explorations remained constant.

**Conclusion:**

According to the postulation, a great decrease in the need of trauma operations was observed during the peak of COVID-19 pandemic. In the future, in case similar public restrictions are ordered, the spared resources could be deployed to other clinical areas. However, the need of pediatric surgical emergencies held stable during the COVID-19 restrictions.

## Introduction

The COVID-19 outbreak of 2019 was declared a pandemic by the World Health Organization (WHO) on 11 March 2020 (1). It carries higher morbidity and mortality compared to seasonal influenza (2). Therefore, significant recommendations and restrictions to “flatten the curve” have been implemented by the governments worldwide, aiming to reduce the burden on healthcare systems (3). In Finland, this has included school closures from 17 March to 14 May 2020. Furthermore, all professional and amateur sport events, as well as trainings, were canceled. In addition, gatherings of more than ten people were not allowed and “social distancing” was recommended (4). Although COVID-19 is usually a mild disease in pediatric population (5), the implemented restrictions have significantly reduced after-school and sporting activities.

Christey et al. (6) reported a significant reduction in all trauma patients after national lockdown for COVID-19 in New Zealand. Bram et al. (7) reported similar reduction in all pediatric fractures during school closures in the United States when numbers were compared with corresponding time period from previous years. It remains unclear, however, if the pandemic has influenced the number of other emergency operations in children.

The aim of our multi-center study was to assess the incidence of pediatric emergency operations related to fractures, appendicitis, and scrotal exploration in all five tertiary University centers in Finland during the COVID-19 pandemic. In line with the previous studies, we anticipated reduced numbers of trauma operations compared to the previous years. In addition, we aimed to assess the injury types related to the trauma and hypothesized that sport-related injuries would have reduced most significantly. We hypothesized that COVID-19 would not have significant effect on the frequency of non-trauma-related emergency operations but we expected to see delays in seeking care.

## Material and Methods

There are five tertiary centers for pediatric surgery and orthopedics in Finland and the data were collected individually at each hospital providing nationwide coverage. Emergency operations were identified using the operating theater management software (Centricity Opera 4.5, GE Healthcare, Barrington, IL, USA or Orbit 5, EVRY Healthcare Systems AB, Kristianstad, Sweden). First, all pediatric (aged above 16 years) emergency operations performed between 1 March and 31 May were identified covering years from 2017 to 2020. Subsequently, emergency operations not related to fractures, appendicitis, or scrotal exploration were excluded. The influence of COVID-19 pandemic was assessed by comparing the monthly incidence of above-mentioned operations in 2020 with corresponding time periods from three previous years.

### Statistical Analysis

Two sample *t*-tests were utilized to analyze differences in monthly operation volumes between prepandemic and pandemic time periods. A significance level of *p* ⩽ 0.05 (two-tailed) was set. Analyses were performed using JMP Pro, version 13.1.0 for Windows (SAS Institute, Inc., Cary, NC, USA).

### Ethical Considerations

The approval of the Institutional Review Boards was obtained individually at each university hospital. Every university collected their data independently and only the anonymized, final numbers were combined.

## Results

We identified 1755 patients undergoing emergency operations during our study period in five tertiary hospitals. This included 1376 fracture operations during prepandemic years and 379 patients during spring 2020. In total, pediatric trauma operations decreased by 24% during the COVID-19 pandemic.

The frequency of all fracture operations reduced most significantly (31%) during March and April 2020 (*p* = 0.03). In May, the reduction was only 13% and not statistically significant. A considerable reduction was observed in lower limb fractures requiring operative treatment, *p* = 0.006 (Table 1). When analyzing fracture mechanisms, school or daycare fractures were significantly less common during the COVID pandemic (*p* < 0.001). Also, organized sport–related fractures were significantly reduced (*p* = 0.001). There was no change in fractures related to trampoline, traffic, and non-organized sports (Table 2).

**Table 1:** Comparison of trauma surgery rates between prepandemic and pandemic months.

	Prepandemic (2017–2019) monthly volume per center Median (range)	Pandemic (2020) monthly volume per center Median (range)	*p*
Trauma surgery			
March	19 (10–34)	7 (4–23)	0.03
April	21 (7–37)	13 (11–30)	
May	29 (9–51)	21 (10–44)	0.56
Upper limb trauma			
March	14 (6–24)	4 (2–17)	0.13
April	12 (4–25)	9 (9–22)	
May	22 (8–39)	17 (9–26)	0.19
Lower limb trauma			
March	7 (3–14)	3 (0–9)	0.006
April	6 (1–12)	4 (1–6)	
May	6 (0–14)	4 (1–15)	0.94
Sex (M/F)	63%/37%	64%/36%	0.74

M: male; F: female.

**Table 2:** Comparison of differences in fracture etiology between prepandemic and pandemic months.

Etiology	Prepandemic (2017–2019) monthly volume per center Mean (range)	Pandemic (2020) monthly volume per center Mean (range)	*p*
School or daycare	3 (0–10)	0 (0–2)	<0.001
Trampoline	1 (0–5)	2.5 (0–6)	0.25
Sports (organized)	2 (1–9)	0 (0–3)	0.001
Sports (non-organized)	4.5 (0–10)	1.5 (0–10)	0.27
Traffic	1 (0–6)	0 (0–4)	0.48
Other	7.5 (2–17)	5.5 (2–18)	0.85

The frequencies for scrotal exploration and surgery for acute appendicitis were similar during the prepandemic and pandemic era.

There was no change in the age or sex of the children undergoing emergency surgery in four university hospitals. However, in Kuopio University Hospital, the age of the fracture patients was significantly lower during the pandemic months (median 12.2 (range of 0.1–15.9) versus 10.4 (range of 3.1–15.7) years, *p* = 0.013).

## Discussion

The COVID-19 pandemic has affected the world from the first months of 2020. There was a rapid increase in COVID-19 cases in Finland in the beginning of March. The Finnish government instituted the Emergency Act on 16 March and implemented several restrictions for the society to slow the spread of the COVID-19. Borders and schools were closed, and social gatherings were restricted to less than ten people. Schools remained closed and people were recommended to avoid social contact and organized sports from 17 March to 14 May (4). We show in our study how the incidence in the pediatric orthopedic trauma procedures started to decrease with the initiation of restrictions. By May, however, the number of trauma patients returned to normal levels before the restrictions were eased.

Over 70% of the coronavirus positive cases in Finland have been in the Capital region of Helsinki (Uusimaa), which accommodates about 30% of the Finnish population. The Uusimaa region was temporarily isolated from the rest of Finland to restrict viral spread. However, the decline in pediatric trauma operations was observed equally in the whole country. We postulate this to be due to implemented restrictions and recommendations in our society. With only 327 COVID-related deaths by mid-June in Finland compared to harder hit countries such as United States, Italy, France, and the United Kingdom (8), the overall reduction in pediatric trauma patients in our study (24%) was relatively lower as compared to United States, for example, where fracture reduction was 58% (7).

Other specialties have reported significant delays in time-to-presentation for care (9–11). Our cohort, however, did not observe any significant delays in any of the three evaluated operation groups in any hospital district. This is in keeping with the results of a large US study, which also reported significant reduction in the incidence of fractures but no significant increase in time-to-presentation for care (7). We postulate that this may also be a reflection of rather controlled COVID-19 spread in Finland during the spring of 2020 (12).

When looking at trauma mechanisms, we observed significant reductions in school or daycare and organized sport–related injuries. Studies reporting epidemiology on children’s fractures have found that sport and play contribute most fracture events (13). In general, upper limb fractures are more common among children with significant male preponderance (13–15). In the pandemic era, we found a similar distribution, but reduction in operative fracture care was only observed in lower limb fractures. We postulate that the reduction in lower limb fractures may be caused by the cessation of organized sports, as it has been reported that majority of pediatric injuries related to football, for example, is located in the lower extremity (16). We did not find statistically significant changes in the ages of the patients, except in East Finland, in Kuopio. This might be due to poor snow conditions. In the pandemic year, there was less snow, and this might have an influence on the amount winter sport–related fractures in older children (17).

This study has obvious limitations due its retrospective nature. We only included pediatric patients, who underwent emergency operations, not patients undergoing conservative treatment. Most pediatric fractures can be treated on an outpatient basis, with only a minor proportion requiring hospitalization or observation (14). Therefore, the numbers observed here will not represent the true spectrum of fractures treated during this period.

Despite of the pandemic misfortune, children in Finland have maintained their mobility while adhering to social distancing. Team sports have been on hold, but the regular physical activities seem to prevail. Operative treatment of fractures has been estimated to be increasing in Finland previously (18). It was speculated that this may be partially linked to a change in spare-time activities with higher trauma energies. This study period showed an initial decrease in pediatric operative traumas, but this returned to baseline rather quickly. Children are active in all circumstances and we must prepare and provide for adequate resources to deal with possible trauma situations at all times.
